# Case Report: Proximal duodenal mural mass causing extrahepatic biliary obstruction and reactive pancreatic changes in a dog

**DOI:** 10.3389/fvets.2025.1636638

**Published:** 2025-10-08

**Authors:** Jae-Yun Ko, Hee-Myung Park, Min-Hee Kang

**Affiliations:** ^1^Pyeonanhan Animal Hospital, Daejeon, Republic of Korea; ^2^Department of Veterinary Internal Medicine, College of Veterinary Medicine, Konkuk University, Seoul, Republic of Korea; ^3^Department of Bio-animal Health, Jangan University, Gyeonggi-do, Republic of Korea

**Keywords:** sterile abscess, duodenal mural mass, biliary obstruction, pancreatitis, exploratory laparotomy

## Abstract

A 10-year-old castrated male Yorkshire Terrier was referred for acute vomiting and inappetence. Blood tests revealed elevated hepatobiliary and pancreatic enzymes. Ultrasonography identified a hypoechoic mural mass in the cranial duodenum with concurrent dilation of the common bile duct and hypoechoic changes in the pancreas. Fine needle aspiration cytology demonstrated numerous neutrophils without bacteria or neoplastic cells. No pathogens were identified on cytology, histopathology, or culture, raising the possibility of a sterile abscess or a necrotic inflammatory lesion. Computed tomography revealed a well-defined, fluid-attenuating, duodenal mural lesion located near the major duodenal papilla. Surgical exploration and drainage were performed. Histopathology showed marked neutrophilic and macrophagic infiltration in the duodenal muscularis layer. Based on clinical, imaging, and histopathologic findings, the dog was diagnosed with a duodenal mural lesion possibly representing a sterile abscess or necrotic inflammatory mass, associated with extrahepatic biliary obstruction and reactive secondary pancreatitis. Clinical signs improved following surgical treatment, and no recurrence was observed during the two-month follow-up period.

## Introduction

Focal mural lesions of the duodenum are rarely reported in veterinary clinical practice. Because of their rarity and non-specific clinical manifestations, they may be challenging to recognize, and definitive diagnosis requires cytologic or histopathologic sampling (1, 2). Such lesions may result from inflammatory, infectious, neoplastic, or traumatic processes and can appear as focal or diffuse thickening, cavitary lesions, or mass-like structures within the intestinal wall (3, 4). Rare cases of idiopathic intramural hematoma have also been reported in dogs, primarily affecting the jejunum, and support the inclusion of mural hematomas in the differential diagnosis of non-specific gastrointestinal symptoms (5). Among these, intramural duodenal abscesses (IDA) are particularly rare. In dogs, reported cases of IDA have typically been associated with mucosal trauma or migration of foreign bodies (6, 7). In contrast, spontaneous IDA without a clear mechanical cause or mucosal disruption is exceedingly rare and remains poorly understood.

The anatomical complexity of the canine cranial duodenum further complicates both clinical presentation and diagnostic evaluation. This region includes the major duodenal papilla, where the common bile duct and pancreatic duct merge and enter the intestinal lumen, making it a critical junction in the pathophysiology of biliary and pancreatic disorders (8). Lesions in this area may exert physical pressure on the ducts or cause functional obstruction secondary to localized inflammation, leading to extrahepatic biliary obstruction (EHBO) and reactive pancreatic changes (9, 10). Clinical signs such as vomiting, abdominal pain, anorexia, and diarrhea are frequently observed in these conditions. However, these signs are non-specific and commonly overlap among gastrointestinal, hepatobiliary, and pancreatic diseases, which interferes early diagnosis, particularly in the absence of overt icterus or clear ductal dilation on imaging (11).

Abdominal ultrasonography is commonly used as the initial diagnostic modality for assessing suspected duodenal mural lesions due to its noninvasive nature and ability to detect structural changes in the intestinal wall. However, its capacity to delineate the precise anatomical relationships between mural abnormalities and adjacent structures such as the bile and pancreatic ducts is often limited, particularly in cases involving deep-seated or poorly vascularized lesions (1, 2). Fine needle aspiration (FNA) may provide useful cytologic information, but its sensitivity for gastrointestinal mural lesions is limited, especially in differentiating inflammation from neoplasia. Therefore, histopathology is usually required for a definitive diagnosis. Yet, distinguishing between sterile and septic inflammation remains challenging, and prior administration of antimicrobials can reduce the diagnostic yield by obscuring cytological or microbiological findings (8). In such cases, contrast-enhanced computed tomography (CT) offers superior spatial resolution and can enhance the characterization of mural lesions and their relationship to surrounding ductal anatomy (8). Despite these combined approaches, the underlying cause and clinical relevance of mural lesions may remain uncertain, and histopathologic evaluation is often required to achieve a definitive diagnosis, especially when imaging and cytology provide inconclusive or conflicting results (12).

To the authors’ knowledge, no previous veterinary report has described a canine IDA located near the major duodenal papilla and concurrently associated with biliary obstruction and reactive pancreatic involvement in the absence of foreign material, neoplasia, or perforation. This case illustrates the diagnostic challenges posed by mural duodenal lesions at anatomically critical sites and supports their inclusion in the differential diagnosis of dogs with overlapping gastrointestinal, biliary, and pancreatic signs.

## Case description

### Case presentation and diagnostic investigations

A 10-year-old, castrated male Yorkshire Terrier (4.22 kg) was referred for evaluation of acute vomiting and dark reddish-brown diarrhea consistent with melena. The clinical signs were preceded by a 4-day history of lethargy and inappetence. The referring veterinarian had initiated empirical treatment with antibiotics, fresh frozen plasma, and antiemetics under the presumptive diagnosis of acute pancreatitis. However, the dog showed no clinical improvement with supportive therapy.

Upon presentation, the dog exhibited yellowish mucoid diarrhea, weight loss, and marked pain on palpation of the cranial abdomen. Hematologic evaluation revealed severe leukocytosis (37.72 × 10^9^/L; reference interval [RI]: 6–17 × 10^9^/L) characterized by a stress leukogram. Serum biochemistry revealed elevated hepatobiliary enzymes, including alkaline phosphatase (ALP, 335 U/L; RI: 15–127 U/L), *γ*-glutamyltransferase (GGT, 7.2 U/L; RI: 0–6 U/L), and total bilirubin (8.38 μmol/L; RI: 0–6.84 μmol/L). Pancreatic enzymes were also markedly elevated, with lipase at 768 U/L (RI: 0–500 U/L) and amylase at 2,297 U/L (RI: 185–700 U/L). Hyperproteinemia was observed (total protein 80.0 g/L; RI: 54.0–74.0 g/L). Albumin and globulin were mildly increased (albumin 44 g/L; RI: 29–42 g/L; globulin 36 g/L; RI: 23–35 g/L), with an A/G ratio of 1.2, consistent with proportional elevation rather than selective increase. Electrolyte levels were within normal limits, and urinalysis showed no abnormalities.

Initial stabilization was instituted immediately. An intravenous catheter was placed, and balanced isotonic crystalloids were administered at maintenance with titrated boluses as indicated. Antiemetic therapy (maropitant; 1 mg/kg SC every 24 h; Cerenia®, Zoetis, USA), analgesia (tramadol; 4 mg/kg SC every 12 h; Tridol®, Youhan Pharm, South Korea), and fasting with aspiration precautions were initiated, and vital parameters were continuously monitored to ensure hemodynamic stability. Diagnostic investigations were undertaken once the patient was stable.

**Figure 1:**
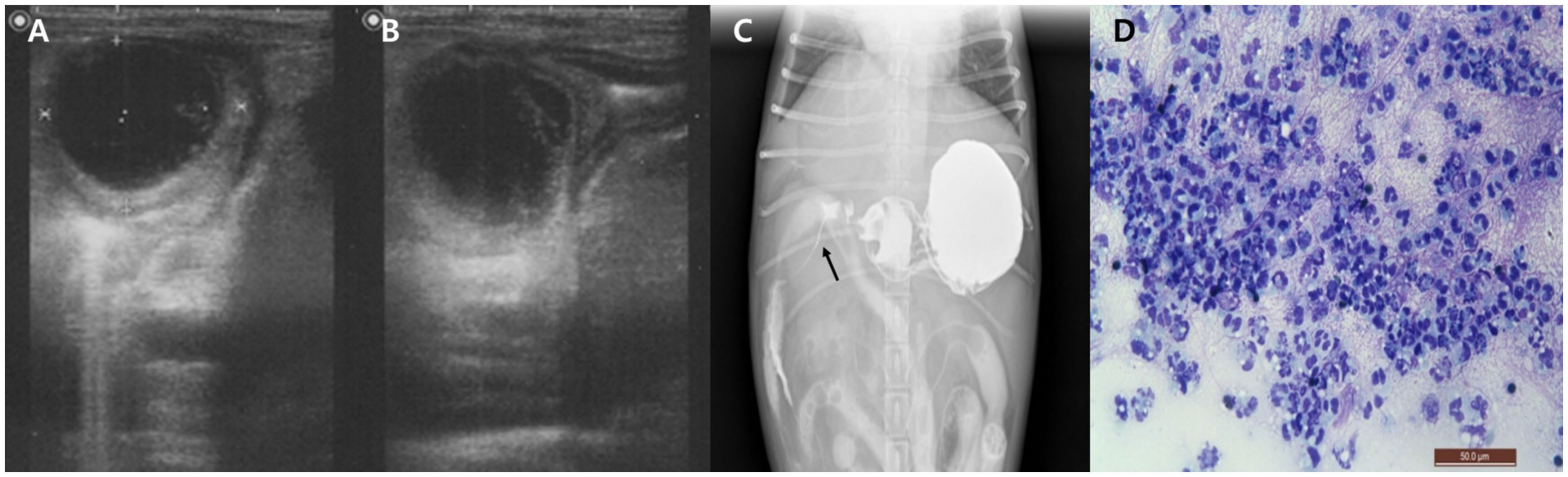
Imaging and cytological findings of an intramural duodenal abscess in a dog. **(A,B)** Abdominal ultrasonography showing a well-demarcated, round, hypoechoic mass (25.3 × 26.1 mm) within the cranial duodenal wall with mild mural thickening. **(C)** Positive contrast radiograph using oral barium sulfate demonstrates narrowed contrast passage with a thread-like appearance at the proximal duodenum (arrow). **(D)** Cytological smear of the duodenal lesion obtained via ultrasound-guided fine needle aspiration reveals abundant non-degenerate neutrophils without evidence of bacteria or neoplastic cells. Diff-Quik stain, 400×, scale bar = 50 μm.

Right lateral and ventrodorsal abdominal radiographs showed increased soft tissue opacity in the right cranial abdomen and hepatomegaly. Ultrasonography identified a well-demarcated, hypoechoic, round mass (25.3 × 26.1 mm) within the cranial duodenal wall, accompanied by mild mural thickening (5.1 mm) and absence of detectable vascular flow on Doppler imaging (Figures 1A,B). The pancreas appeared diffusely hypoechoic and edematous. The gallbladder was mildly distended, and the common bile duct (CBD) was dilated, although its maximum diameter was not recorded. No abdominal effusion was identified on ultrasonographic examination. Despite these findings, the dog remained non-icteric on physical examination, and serum bilirubin levels were only mildly elevated, suggesting partial or early-stage biliary obstruction.

After stabilization, to assess for potential duodenal luminal narrowing, a positive contrast study was performed using oral barium sulfate (30% w/w, 12 mL/kg; Solotop®, Taejoon Pharm, South Korea). Although oral contrast administration in vomiting patients carries inherent risks, the procedure was carried out cautiously and no complications occurred in this case. Radiographic and fluoroscopic images revealed narrowed contrast flow with a thread-like appearance at the proximal duodenum (Figure 1C). To further characterize the lesion, ultrasound-guided FNA of the duodenal mass was conducted. Cytological examination of the stained smear showed abundant non-degenerate neutrophils without evidence of bacteria or neoplastic cells (Figure 1D). Although frank purulent fluid was not aspirated, the cytologic findings supported an inflammatory process. The aspirated material was subjected to aerobic and anaerobic bacterial culture. All cultures yielded no growth, possibly reflecting prior antibiotic therapy or a sterile inflammatory lesion such as an aseptic abscess. Blood cultures performed at the time of presentation and repeated 24 h later were both negative.

**Figure 2:**
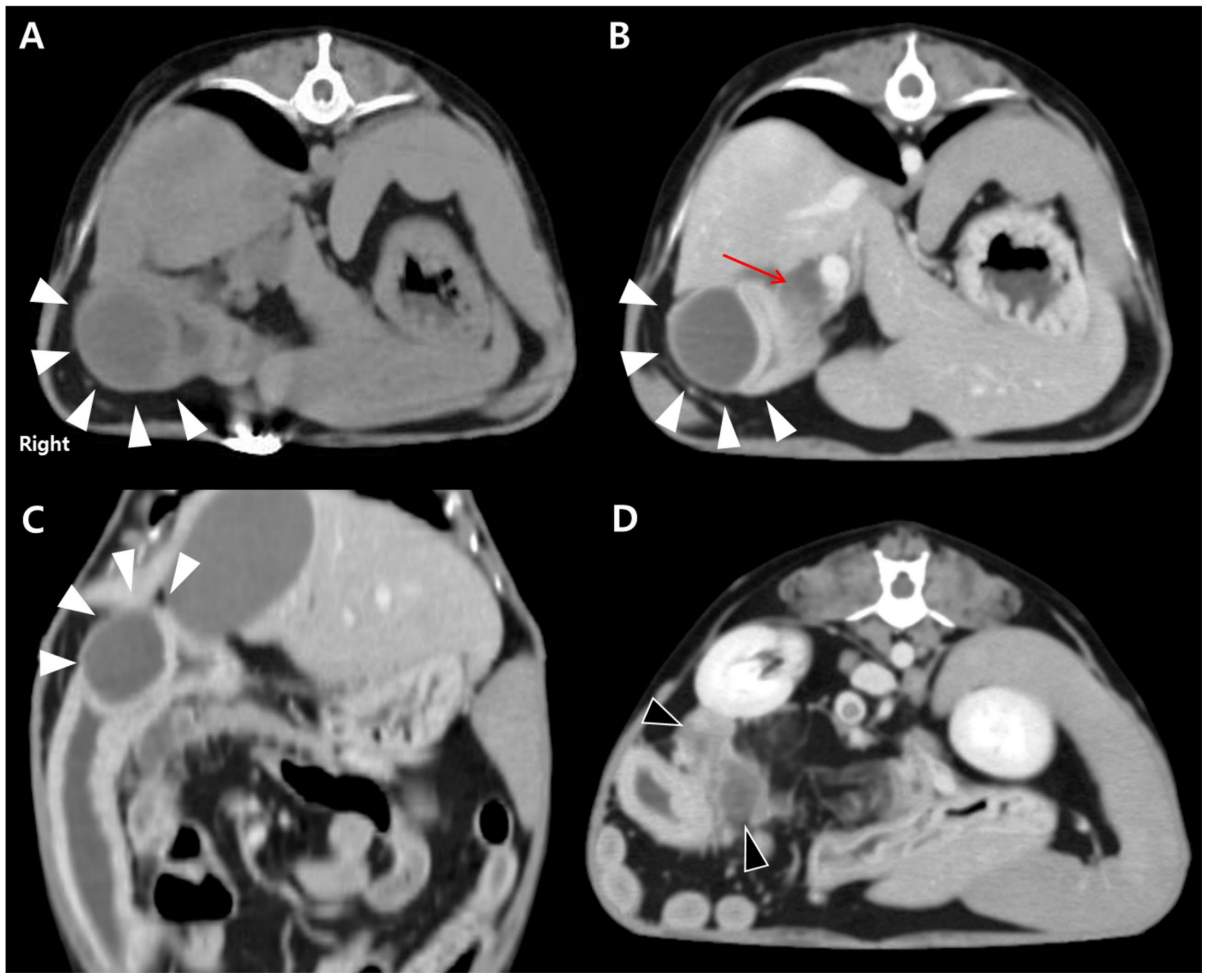
CT findings of an intramural duodenal abscess and associated biliary and pancreatic changes in a dog. **(A)** Transverse pre-contrast CT image showing a well-demarcated, fluid-attenuating, round mural lesion in the proximal descending duodenum (white arrowheads). **(B)** Transverse post-contrast image demonstrating peripheral rim enhancement of the lesion (white arrowheads) with central hypoattenuation. Marked dilation of the biliary tree, including the cystic, hepatic, and common bile ducts, is evident (red arrow). **(C)** Dorsal post-contrast image further delineating the lesion (white arrowheads) adjacent to the major duodenal papilla. **(D)** Transverse post-contrast image showing diffuse hypoattenuating changes in the pancreas (black arrowheads), consistent with reactive pancreatic involvement.

For further evaluation, contrast-enhanced CT was performed after stabilization under general anesthesia with endotracheal intubation and standard monitoring (ECG, SpO₂, capnography, and non-invasive blood pressure). A well-defined, encapsulated, fluid-attenuating mural lesion was identified in the proximal descending duodenum, near the major duodenal papilla and the opening of the CBD (Figures 2A–C). The lesion exhibited peripheral contrast enhancement and central hypoattenuation. CT also revealed CBD dilation (6.15 mm) and pancreatic changes characterized by diffuse hypoattenuation, which was considered compatible with edema (Figures 2B,D). Pancreatic lipase immunoreactivity (PLI) was not assessed in this case. The suspicion of secondary pancreatic involvement was based on the combination of elevated serum amylase and lipase activities, which have limited sensitivity for pancreatitis, together with contrast-enhanced CT findings of diffuse pancreatic hypoattenuation compatible with edema. Taken together, these findings supported the diagnosis of an inflammatory duodenal mural mass, considered the likely cause of secondary extrahepatic biliary obstruction and reactive pancreatic involvement.

In-hospital medical therapy included fresh frozen plasma (10 mL/kg IV every 12 h; empirical administration at the referring clinic was continued early in hospitalization), amoxicillin–clavulanic acid (12.5 mg/kg PO every 12 h; Clavamox®, Zoetis, USA), and metronidazole (15 mg/kg IV every 12 h; Flasinyl®, HK inno. N, South Korea). Antiemesis with maropitant and analgesia with tramadol were continued as initiated during stabilization. Partial parenteral nutrition was initiated due to persistent vomiting, using a peripheral intravenous line. The formulation consisted of an amino acid–dextrose solution, covering approximately 40% of the estimated daily energy requirement. Administration was gradually titrated over 24 h with continuous monitoring of tolerance and metabolic parameters. By day 5 of hospitalization, serum pancreatic enzyme levels had normalized. However, hepatobiliary enzymes continued to rise (ALP increased from 335 to 416 U/L; GGT from 7.2 to 27 U/L), and clinical signs remained unimproved. Serial ultrasonography performed over a 7-day period showed no significant changes in the duodenal mass, gallbladder, or CBD.

**Figure 3:**
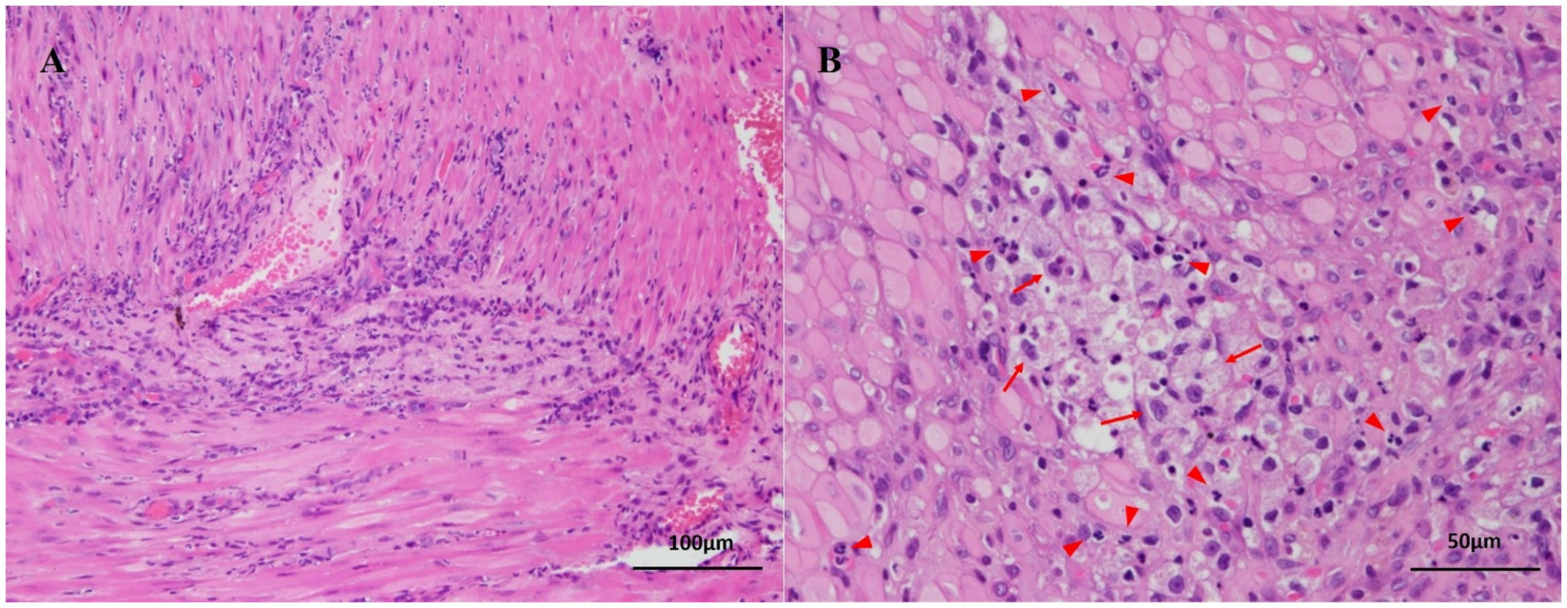
Histopathological findings of an intramural duodenal abscess in a dog. **(A)** Diffuse infiltration of numerous neutrophils is observed within the muscular layer of the duodenal wall, without involvement of the mucosa or serosa. No neoplastic cells or vascular thrombosis are present. Hematoxylin and eosin stain; bar = 100 μm. **(B)** Chronic inflammation is characterized by numerous infiltrating neutrophils (red arrowheads) and macrophages containing phagocytized neutrophils (red arrows). A small amount of necrotic debris is present in the central region of the lesion. Hematoxylin and eosin stain; bar = 50 μm.

Due to the lack of response to medical management, exploratory laparotomy was performed. On surgical exploration, the serosal surface of the duodenal mass was carefully incised to allow controlled drainage of purulent material, thereby reducing mural tension and minimizing the risk of inadvertent perforation. The cavity was aspirated and lavaged to limit peritoneal contamination, and an excisional biopsy of the duodenal lesion was subsequently obtained. Histopathological examination revealed dense infiltration of macrophages and numerous neutrophils within the muscular layer, consistent with a diagnosis of IDA (Figure 3). No evidence of neoplasia, mucosal ulceration, or vascular thrombosis was noted. A small amount of necrotic debris was observed in the central region of the muscularis layer (Figure 3B), without involvement of the mucosa or serosa. Following surgery, the dog recovered well and showed steady clinical improvement over the following days. Postoperative management included continuation of broad-spectrum antibiotics, analgesia, and antiemetic therapy, together with intravenous fluid support and careful monitoring for signs of peritonitis or dehiscence. Nutritional support was gradually transitioned from parenteral to enteral feeding as vomiting resolved. The patient was discharged 3 days postoperatively without complications. Stool consistency improved progressively, with normal fecal consistency observed by 1 week post-discharge. The dog remained clinically stable without recurrence of gastrointestinal signs throughout the two-month follow-up period.

## Discussion

This case highlights the diagnostic complexity and clinical relevance of IDA in dogs, particularly when the lesion is positioned in a region with critical anatomical and functional associations. The lesion in this case developed within the proximal descending duodenum, in close proximity to the major duodenal papilla. Lesions at this junction can affect multiple systems via mechanical compression or localized inflammation. The case presented here was further characterized by EHBO and reactive pancreatic involvement, despite the absence of foreign bodies, neoplasia, or mucosal ulceration. These features indicate the importance of considering mural duodenal disease in the differential diagnosis for dogs with overlapping signs involving the gastrointestinal, biliary, and pancreatic systems.

Among the gastrointestinal signs, the patient’s fecal characteristics also warrant consideration. The dog initially showed melena but later developed mucoid diarrhea after referral. Mucoid feces are not pathognomonic for small intestinal disease and are more frequently associated with colonic irritation or inflammation (13). In this case, the change in fecal character may suggest secondary colonic involvement, potentially influenced by mucosal irritation or dysbiosis following antibiotic therapy (14). Nonetheless, the clinical and imaging findings indicated that the small intestine was the critical site of disease, and diagnostic and therapeutic decisions were primarily directed to this region.

Intramural duodenal abscesses are rarely reported in veterinary medicine. Most previously described cases involved penetrating foreign bodies or trauma to the intestinal wall (6, 7). In contrast, the present case appeared to arise spontaneously, without evidence of mechanical injury or ulceration, suggesting a sterile or idiopathic origin. The lesion was encapsulated and restricted to the muscularis layer, and no causative organisms were identified, even after repeated blood and tissue cultures. This pattern supports a non-neoplastic inflammatory process with potential sterile etiology.

From a diagnostic standpoint, ultrasonography and cytology suggested inflammation but were inconclusive regarding ductal relationships and etiology. Culture negativity possibly resulting from antecedent antimicrobials (8) did not exclude a walled-off intramural process.

A limited oral contrast study suggested partial obstruction but provided little additional information beyond cross-sectional imaging. In contrast, contrast-enhanced CT was pivotal in characterizing the lesion and its relationship to the papilla. The CT findings were not only consistent with a mature abscess but also demonstrated ductal and pancreatic changes that explained the dog’s clinical syndrome. This underscores the unique diagnostic value of CT in duodenal mural lesions, particularly near the papilla, where secondary biliary and pancreatic involvement can be overlooked with conventional imaging (1, 4, 15).

PLI was not evaluated in this case. Serum amylase and lipase were elevated; however, these enzymes have limited sensitivity for pancreatitis and may remain normal even in histologically confirmed cases (16). Therefore, the suspicion of secondary pancreatic involvement was based not on enzyme activities alone but on their elevation together with contrast-enhanced CT findings of diffuse pancreatic hypoattenuation consistent with edema. Similar cases have described obstructive or reactive pancreatitis associated with lesions near the duodenal papilla, even in the absence of direct pancreatic pathology (11). Histopathology confirmed an intramural inflammatory lesion confined to the muscularis, without neoplasia or mucosal disruption. While such a cellular pattern may also occur in infectious processes, including mycobacterial or Leishmania-associated granulomatous inflammation, these etiologies were considered unlikely in this case given the absence of systemic signs, negative culture results, and regional epidemiology.

Several differential diagnoses were considered, including intestinal neoplasms (such as lymphoma and leiomyosarcoma), hematoma, granuloma, and fibrogranulomatous eosinophilic sclerosing fibroplasia (2, 3, 5, 15). Imaging features helped narrow the differential, but a definitive diagnosis was only achieved through histological assessment.

Following unsuccessful medical management, surgical drainage with excisional biopsy served both diagnostic and therapeutic purposes, with uneventful recovery. This outcome supports the therapeutic role of early surgical intervention in selected cases where mural lesions cause persistent clinical signs or are suspected to compress ductal structures (17).

FFP was administered empirically early in hospitalization when pancreatitis was suspected. At that time, there were no documented coagulation abnormalities, hypoalbuminemia, or active bleeding, and coagulation monitoring was not performed. Historically, FFP has been administered in pancreatitis cases for theoretical benefits such as albumin supplementation or *α*-macroglobulin replacement, and earlier surveys listed triggers including persistent vomiting (18). However, more recent analyses indicate a marked decline in such empirical use, with transfusions now primarily reserved for confirmed coagulopathies, and studies in dogs with pancreatitis have shown no survival benefit and even higher mortality among plasma recipients (19, 20). Thus, the administration of FFP in this case should be interpreted as a limitation and does not represent standard therapy.

Despite the clinical and diagnostic insights offered by this case, certain limitations should be acknowledged. First, PLI was not measured, which restricted confirmation of pancreatitis and limits diagnostic certainty. Although the imaging and serum enzyme findings were suggestive of secondary pancreatic involvement, histopathologic confirmation was not obtained. Pancreatic tissue sampling might have provided additional academic insight, but it is not routinely indicated in clinical cases, and the available evidence was considered sufficient to support the interpretation in this dog. Additionally, as this is a single case report, broader conclusions cannot be drawn, and the proposed pathophysiologic mechanism and clinical associations should be interpreted cautiously. Further studies with larger case numbers are warranted to validate these observations.

In conclusion, this case suggests that mural duodenal abscesses, although rare, can have significant secondary effects on adjacent biliary and pancreatic systems, particularly when located near the major duodenal papilla. From a clinical perspective, this case emphasizes that veterinarians should include mural duodenal lesions in the differential diagnosis of dogs presenting with concurrent gastrointestinal, hepatobiliary, or pancreatic signs. A multimodal approach, including ultrasonography, cross-sectional imaging, and histopathology, is essential for accurate diagnosis and effective management.

## Data Availability

The original contributions presented in the study are included in the article/supplementary material, further inquiries can be directed to the corresponding author.
